# Cytokine profiles as markers of disease severity in sepsis: a multiplex analysis

**DOI:** 10.1186/cc5783

**Published:** 2007-04-21

**Authors:** Fernando A Bozza, Jorge I Salluh, André M Japiassu, Marcio Soares, Edson F Assis, Rachel N Gomes, Marcelo T Bozza, Hugo C Castro-Faria-Neto, Patrícia T Bozza

**Affiliations:** 1 ICU, Instituto de Pesquisa Clínica Evandro Chagas, Fundação Oswaldo Cruz, Av Brasil 4365, Rio de Janeiro, Brazil; 2 ICU, Hospital Universitário Clementino Fraga Filho, Universidade Federal do Rio de Janeiro, Rio de Janeiro, Brazil; 3 ICU, Hospital Barra D'Or, Av. Ayrton Senna, 2541, Rio de Janeiro, 22775-001, Brazil; 4 ICU, Instituto Nacional do Câncer, Rio de Janeiro, Brazil; 5 ICU, Hospital Quinta D'Or, R. Almirante Baltazar 435, Rio de Janeiro, 20941-150, Brazil; 6 Laboratório de Imunofarmacologia, Departamento de Fisiologia e Farmacodinâmica, IOC, Fundação Oswaldo Cruz, Av Brasil 4365, Rio de Janeiro, 21045-900, Brazil; 7 Departamento de Imunologia, Instituto de Microbiologia, Universidade Federal do Rio de Janeiro, Rio de Janeiro, RJ, 21941-590, Brazil

## Abstract

**Introduction:**

The current shortage of accurate and readily available, validated biomarkers of disease severity in sepsis is an important limitation when attempting to stratify patients into homogeneous groups, in order to study pathogenesis or develop therapeutic interventions. The aim of the present study was to determine the cytokine profile in plasma of patients with severe sepsis by using a multiplex system for simultaneous detection of 17 cytokines.

**Methods:**

This was a prospective cohort study conducted in four tertiary hospitals. A total of 60 patients with a recent diagnosis of severe sepsis were included. Plasma samples were collected for measurement of cytokine concentrations. A multiplex analysis was performed to evaluate levels of 17 cytokines (IL-1β, IL-2, IL-4, IL-5, IL-6, IL-7, IL-8, IL-10, IL-12, IL-13, IL-17, interferon-γ, granulocyte colony-stimulating factor [G-CSF], granulocyte-macrophage colony-stimulating factor, monocyte chemoattractant protein [MCP]-1, macrophage inflammatory protein-1 and tumour necrosis factor-α). Cytokine concentrations were related to the presence of severe sepsis or septic shock, the severity and evolution of organ failure, and early and late mortality.

**Results:**

Concentrations of IL-1β, IL-6, IL-7, IL-8, IL-10, IL-13, interferon-γ, MCP-1 and tumour necrosis factor-α were significantly higher in septic shock patients than in those with severe sepsis. Cytokine concentrations were associated with severity and evolution of organ dysfunction. With regard to the severity of organ dysfunction on day 1, IL-8 and MCP-1 exhibited the best correlation with Sequential Organ Failure Assessment score. In addition, IL-6, IL-8 and G-CSF concentrations during the first 24 hours were predictive of worsening organ dysfunction or failure of organ dysfunction to improve on day three. In terms of predicting mortality, the cytokines IL-1β, IL-4, IL-6, IL-8, MCP-1 and G-CSF had good accuracy for predicting early mortality (< 48 hours), and IL-8 and MCP-1 had the best accuracy for predicting mortality at 28 days. In multivariate analysis, only MCP-1 was independently associated with prognosis.

**Conclusion:**

In this exploratory analysis we demonstrated that use of a multiple cytokine assay platform allowed identification of distinct cytokine profiles associated with sepsis severity, evolution of organ failure and death.

## Introduction

Identifying high-risk patients with sepsis is a great challenge in the care of critically ill patients [1,2]. Most decisions in patients with severe sepsis are based on clinical and laboratory data with poor accuracy [3]. Therefore, efforts to enhance knowledge of the pathophysiology of systemic inflammation and to identify more accurate predictors of prognosis are important [4,5]. Ideally, biomarkers should provide valuable information regarding diagnosis and prognosis, and should permit one to monitor the patient's response to treatment [6].

Although for decades prognostication in critically ill patients has been achieved by quantifying the degree of physiological derangement, such indices do not take into account the patient's specific alteration in immune status. Recently, a new clinical staging system for sepsis was proposed [7], and in this model identification of biomarkers that play central roles in the pathogenesis of sepsis is crucial [8]. Cytokines are proteins that are secreted by components of the innate and adaptive immune systems, and they act as effectors or modulators of inflammatory response, which in turn play prominent roles in the development of sepsis [9].

New technologies for cytokine quantification have recently been developed [10-12]. Among those, the multiplex analysis system, which uses a combination of fluorescently dyed microspheres associated with a two-laser flow cytometry based system [13], permits the simultaneous analysis of up to 100 different biomolecules (proteins, peptides, or nucleic acids) in a single microplate well using small samples [14]. Recent studies indicate that this multiplex analysis system could be used to measure cytokine concentrations in lipopolysaccharide-stimulated human plasma samples, demonstrating that it would be feasible to detect and quantify cytokines and other potential biomarkers in a complex milieu such as human septic plasma [15].

The aim of the present study was to determine the cytokine profile in plasma of patients with severe sepsis by using a multiplex system that permits simultaneous detection of 17 cytokines. We conducted an exploratory analysis and found that the multiple cytokine assay platform was able to identify distinct cytokine profiles associated with sepsis severity, evolution of organ failure and death.

## Materials and methods

### Patients

Our ethics committee approved the present study, and signed informed consent was obtained from all participants. We prospectively included 60 patients who, based on strong suspicion of infection, were admitted to the medical-surgical intensive care units at the Hospital Universitário Clementino Fraga Filho-UFRJ, Hospital Espanhol, Hospital Barra D'or and Hospital Quinta D'or (Rio de Janeiro, Brazil). Patients were eligible for inclusion if they fulfilled criteria for systemic inflammatory response syndrome and had an obvious source of infection. Systemic inflammatory response syndrome, severe sepsis and septic shock were defined in accordance with the American College of Chest Physicians/Society of Critical Care Medicine Consensus Conference [16]. Severity of illness was assessed by calculating the Acute Physiology and Chronic Health Evaluation (APACHE) II score for the first 24 hours [17] and the Sequential Organ Failure Assessment (SOFA) score [18] on days 1 and 3. Based on variations in the SOFA score between days 1 and 3, patients were categorized as 'improved' if the SOFA category decreased by 1 point or more, or 'not improved' if the category remained the same or increased by 1 point or more (modified from Levy an coworkers [19]). Patients were excluded in case of death within six hours of admission or if they were under 18 years old. None of the patients received anti-inflammatory agents, corticosteroids, or other sepsis-modifying agents before enrolment or during the study period. Early mortality was defined as death occurring during the first 48 hours. The main outcome measure of interest was 28-day mortality.

### Multiplex cytokine assay

Blood samples were collected between 10:00 and 12:00 hours using an arterial line or a peripheral vein. Blood was put on ice and plasma was collected by centrifugation at 800 *g *for 15 min at 4°C, aliquoted and stored at -70°C until analysis. A multiplex cytokine kit (IL-1β, IL-2, IL-4, IL-5, IL-6, IL-7, IL-8, IL-10, IL-12, IL-13, IL-17, IFN-γ, granulocyte colony-stimulating factor [G-CSF], granulocyte-macrophage colony-stimulating factor, monocyte chemoattractant protein [MCP]-1, macrophage inflammatory protein-1 and tumour necrosis factor [TNF]-α) was obtained and the assay performed in accordance with the manufacturer's instructions (Bio-Rad, Hercules, CA, USA).

In brief, the appropriate cytokine standards and samples (50 μl), diluted in plasma dilution buffer, were added to wells of a filtered plate. The samples were incubated with 50 μl of the antibody-coupled microsphere set (2,000 beads/well) at room temperature for 30 min on a plate shaker (set to 300 rpm) in the dark and filter washed three times with 100 μl wash buffer. Freshly diluted secondary/detection antibody (25 μl/well) was added to the wells and then incubated at room temperature on a plate shaker for 30 min in the dark and filter washed three times with 100 μl wash buffer. Fifty microlitres of streptavidin-PE (16 μg/ml in assay buffer) was added to the wells, and incubation at room temperature continued for the first 10 min on a plate shaker. Unbound analytes were filtered through the wells using the vacuum manifold and the bound beads were washed three times with 100 μl/wash buffer. After the last wash step, 125 μl of assay buffer was added to each well and the plate placed for 1 min on a plate shaker set at 500 rpm and then for 3 min at the reduced speed of 300 rpm.

Fifty microlitres of sample was analyzed on the Bio-Plex system (Bio-Rad) in accordance with the manufacturer's instructions. Data analyses for all assays were performed using the Bio-Plex Manager software. Cytokine detection using multiplex bead array assays exhibits high degrees of intra-assay (< 10% variation) and inter-assay (10% to 20% variation) precision [13,20]. Cytokine detection by Luminex xMAP technology is comparable to that with enzyme-linked immunosorbent assay (ELISA; correlation coefficient *r *ranges from 0.75 to 0.99) [13,20,21]. Accordingly, when we compared IL-6 detection by Luminex technology and by conventional ELISA (R&D System, Minneapolis, MN, USA) in 31 septic patients, we observed good correlation between the two technologies (*r *= 0.815; *P *< 0.001).

### Statistical analysis

Statistical analyses were performed using SPSS for Windows 10.0 (SPSS Inc., Chicago, IL, USA) and GraphPad Prism version 3.0 for Windows (GraphPad Software, San Diego, CA, USA). Numeric variables are expressed as median (interquartile range) and were assessed using Mann-Whitney *U*-test and Kruskal-Wallis test. Dichotomous variables were analyzed using χ^2 ^and Fisher's exact test (with Yates correction as indicated). Spearman analysis was employed top detect correlations among continuous variables. Receiver operating characteristic (ROC) curves were constructed by plotting the sensitivity versus 1 – specificity, and area under the ROC curve (AUROC) was used to evaluate the ability of each cytokine level to discriminate survivors from nonsurvivors and to predict the evolution of organ dysfunction [22]. Univariate and multivariate logistic regression were used to identify factors associated with hospital mortality. Linearity between continuous variables and the dependent variable was demonstrated using locally weighted scatterplot smoothing (Lowess).

Cytokine concentrations required a log transformation to satisfy the linearity assumption. Variables yielding *P *values below 0.2 by univariate analysis were entered into a forward multivariate logistic regression analysis [23]. Multivariate analysis results were summarized by estimating odds ratios and respective 95% confidence intervals (CIs). The other covariates were entered into the model with critical entry and removal *P *values of 0.05 and 0.1. Effects on covariate coefficients were also considered. Two-tailed *P *values below 0.05 were considered statistically significant.

## Results

### Patients characteristics

Sixty patients were included in this study; 31 (51.7%) patients survived and 29 (48.3%) died. Demographic, clinical and microbiological data for the survivors and nonsurvivors are summarized in Table 1. Patients who died had higher APACHE II and SOFA scores, as expected, compared with survivors. The leading source of infection was the respiratory tract. Micro-organisms were isolated in 50 out of 60 septic patients (83.3%), with a predominance of Gram-negative bacteria (76.0%).

**Table 1:** Patient characteristics

Characteristic	All patients (*n *= 60)	Survivors (*n *= 31)	Nonsurvivors (*n *= 29)
Age (years)^a^	64 (51–75)	55 (48.5–81.50)	64.0 (58.5–73.5)
Gender (male/female)	36/24	19/12	17/12
APACHE II score (points)^a^	20 (17–23)	17.0 (14.5–20.0)	22.0 (20.0–28.0)*
SOFA score on day 1^a^	9 (6–11)	7 (5–9)	11 (8.5–12)*
SOFA score on day 3^a^	7 (5–10)	6 (3–7)	10.5 (8.5–12.5)*
Septic shock	46/60	19/31	27/29
Sites of infection			
Lung	33 (55.00%)	20 (64.52%)	13 (44.83%)
Abdomen	15 (25.00%)	5 (16.13%)	10 (34.48%)
Blood	5 (8.33%)	3 (9.68%)	2 (6.90%)
Other	7 (11.66%)	3 (9.68%)	4 (13.79%)
Microbiological data			
Gram-negative bacteria	37 (61.66%)	18 (66.67%)	19 (82.50%)
Gram-positive bacteria	7 (11.66%)	5 (18.52%)	2 (8.69%)
Polybacterial	4 (6.66%)	2 (7.41%)	2 (8.69%)
Fungi	2 (3.33%)	2 (7.41%)	0 (0%)
Positive cultures	50 (83.33%)	27 (87.10%)	23 (76.00%)
Positive blood cultures	12 (20.00%)	8 (29.63%)	4 (17.39%)

Unless otherwise stated, values are expressed as number or number (%). ^a^Median (interquartile range). *P < 0.05, survivors versus nonsurvivors. APACHE, Acute Physiology and Chronic Health Evaluation; SOFA, Sequential Organ Failure Assessment.

### Cytokine concentrations

When data from all 1,020 assays were analyzed, the multiplex cytokine system was able to detect plasma cytokines in 710 (69.6%) assays. (Here we define 'assay' as each cytokine measured, which was performed on each individual plasma sample.) Concentrations of IL-6, IL-8, IL-10 and macrophage inflammatory protein-1 were detectable in more than 95% of individual assays.

We compared cytokine concentrations among patients with severe sepsis and septic shock, and observed that concentrations of IL-1β, IL-6, IL-7, IL-8, IL-10, IL-13, IFN-α, MCP-1 and TNF-α were significantly increased in septic shock as compared with severe sepsis (Table 2).

**Table 2:** Plasma cytokine concentrations: severe sepsis versus septic shock

Cytokine	Severe sepsis (*n *= 14)	Septic shock (*n *= 46)	*P *value^a^
IL-1β	0.17 (0.00–0.79)	1.22 (0.01–7.33)	0.01
Il-6	1027 (583.1–4854)	5632 (1889–12170)	0.007
IL-7	0.00 (0.00–0.00)	8.475 (0.60–13.56)	< 0.001
IL-8	52.63 (24.16–122.4)	145.3 (74.37–520.2)	0.01
IL-10	2.270 (0.9500–11.72)	27.45 (6.835–116.3)	< 0.001
IL-13	0.27 (0.00–4.61)	7.21 (0.03–19.29)	0.008
IFN-γ	0.0000 (0.00–22.77)	33.10 (0.00–116.7)	0.03
MCP-1	6.295 (0.00–372.2)	753.9 (324.6–1689)	< 0.001
TNF-α	0.00 (0.00–2.78)	14.46 (2.68–47.00)	< 0.001

Values are in pg/ml, and as expressed as mean (range). ^a^Mann-Whitney rank sum test. IL, interleukin; IFN, interferon; MCP, monocyte chemoattractant protein; TNF, tumour necrosis factor.

### Cytokine concentrations and evolution of organ failures

**Figure 1:**
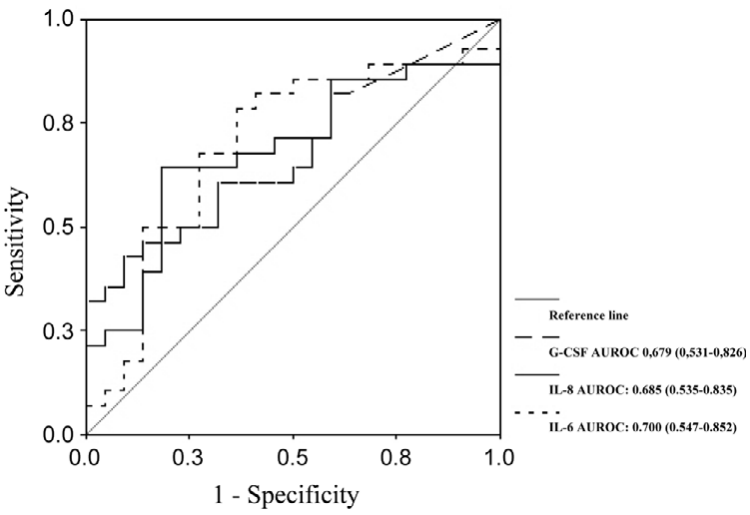
Receiver operating characteristic curve analysis of granulocyte colony-stimulating factor (G-CSF), IL-6 and IL-8 predicting organ dysfunction. Shown are the areas under the receiver operating characteristic curve (AUROCs) for granulocyte colony-stimulating factor (G-CSF), IL-6 and IL-8 predicting failure of organ dysfunction to improve by day 3. The values shown in parentheses are the 95% confidence intervals.

We evaluated the relation of cytokine concentrations to the severity of acute organ dysfunction, as assessed using the SOFA score. We observed a positive correlation between the cytokines IL-1β, IL-6, IL-8, IL-10, MCP-1 and G-CSF, and SOFA score on day 1. The best degrees of correlation were observed for IL-8 and MCP-1 (*r *= 0.50 and 0.43, respectively; *P *< 0.01). In addition, we investigated the ability of cytokines to predict early adverse outcomes, defined as death occurring during the first 48 hours or failure of organ dysfunction to improve by day 3. Nine patients (15%) died within the first 48 hours. IL-1β, IL-4, IL-6, IL-8, MCP-1 and G-CSF had good accuracy for predicting the occurrence of early mortality (< 48 hours). The AUROC for all six cytokines are presented in Table 3. Higher concentrations of IL-6, IL-8 and G-CSF (on day 1) were present in patients whose organ dysfunction worsened or failed to improve by day 3 (Figure 1). As expected, mortality at day 28 was significantly higher in patients whose SOFA score on day 3 did not improve as compared to those whose SOFA score improved (53.6% versus 21.7%; *P *= 0.02).

**Table 3:** Performance of cytokines in predicting early mortality (48 hours)

Cytokine	AUROC (95% CI)	*P *value
IL-8	0.780 (0.621–0.93)	0.012
IL-4	0.767 (0.587–0.94)	0.011
IL-6	0.756 (0.602–0.91)	0.015
MCP-1	0.738 (0.571–0.905)	0.024
G-CSF	0.727 (0.526–0.92)	0.041
IL-1β	0.716 (0.532–0.90)	0.040

AUROC, area under the receiver operating characteristic curve; CI, confidence interval; G-CSF, granulocyte colony-stimulating factor; IL, interleukin; IFN, interferon; MCP, monocyte chemoattractant protein.

### Predictive value of cytokines for severe sepsis 28-day mortality

**Figure 2:**
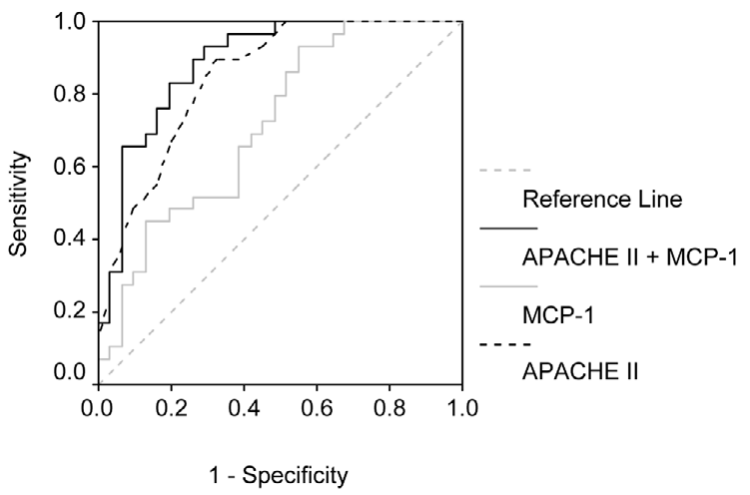
Receiver operating characteristic curves of Acute Physiology and Chronic Health (APACHE) II score, monocyte chemoattractant protein (MCP)-1 and a composite variable (APACHE II + MCP-1) predicting mortality. Shown are areas under receiver operating characteristic curves (AUROCs) for APACHE II score, monocyte MCP-1 and a composite variable (APACHE II + MCP-1), created according to the final model from multivariable analysis (Table 4).

The cytokine plasma concentrations of survivors and nonsurvivors (death by 28 days) are shown in Table 4. Of the 17 cytokines studied, five (IL-1β, IL-4, IL-6, IL-8 and MCP-1) were significantly higher in nonsurvivors. Among the 17 cytokines, only six exhibited AUROC values above 0.6, and discrimination was considered adequate (AUROC > 0.7) only for IL-8 and MCP-1 (Table 5). In multivariate analysis, a base model including APACHE II score and cytokines with AUROC above 0.7 was created. Age (years), IL-6, IL-1β, IL-4 and IL-10 were forced (each variable was forced in separately) into the final model and were not selected. Among the covariates, only MCP-1 and APACHE II score were independently associated with increased mortality (Table 6). The final model, including MCP-1 and APACHE II score, had good discrimination (AUROC = 0.888, 95% CI = 0.804 to 0.971; *P *< 0.001). AUROC of the multivariate model was slightly higher than those of APACHE II score (0.848, 95% CI = 0.752 to 0.944) and MCP-1 (0.715, 95% CI = 0.586 to 0.844) alone (Figure 2). Nonetheless, the 95% CIs were rather wide, resulting in significant overlap of AUROC findings.

**Table 4:** Plasma cytokine concentrations: survivors versus nonsurvivors

Cytokines	Survivors (*n *= 31)	Nonsurvivors (28-day mortality; *n *= 29)	*P *value^a^
IL-1β	0.39 (0.00–3.04)	1.30 (0.22–7.21)	0.050
IL-2	2.80 (0.00–8.10)	3.01 (0.00–7.21)	0.982
IL-4	0.00 (0.00–0.03)	0.84 (0.00–26.28)	0.042
IL-5	1.76 (0.08–6.19)	0.47 (0.00–2.28)	0.067
IL-6	1,957.77 (971.92–6,295.47)	6,254.96 (2,446.01–15,972.40)	0.014
IL-7	1.30 (0.00–14.14)	7.61 (0.60–10.96)	0.297
IL-8	94.69 (20.94–138.35)	281.39 (83.99–773.51)	0.001
IL-10	9.70 (2.00–40.89)	26.92 (5.29–96.58)	0.137
IL-12	1.09 (0.00–40.89)	1.04 (0.00–6.79)	0.958
IL-13	4.69 (0.00–17.90)	3.13 (0.00–12.94)	0.804
IL-17	0.00 (0.00–0.00)	0.00 (0.00–0.20)	0.085
IFN-γ	12.34 (0.00–91.69)	28.71 (0.00–122.94)	0.492
G-CSF	116.00 (12.00–367.00)	423.61 (0.00–2,488.50)	0.336
GM-CSF	0.00 (0.00–21.68)	0.00 (0.00–140.97)	0.221
MIP-1	253.25 (127.49–365.95)	228.41 (140.80–617.30)	0.706
MCP-1	268.36 (0.00–759.30)	757.78 (324.61–1,966.88)	0.004
TNF-α	8.00 (0.00–25.72)	9.04 (2.31–43.28)	0.224

Values are in pg/ml, and as expressed as mean (range). ^a^Mann-Whitney rank sum test. G-CSF, granulocyte colony-stimulating factor; GM-CSF, granulocyte-macrophage colony-stimulating factor; IL, interleukin; IFN, interferon; MCP, monocyte chemoattractant protein; MIP, macrophage inflammatory protein; TNF, tumour necrosis factor.

**Table 5:** Performance of cytokines in predicting 28-day mortality

Cytokine	AUROC (95% CI)	*P *value
IL-8	0.752 (0.626–0.877)	0.001
MCP-1	0.715 (0.586–0.844)	0.004
IL-6	0.684 (0.548–0.820)	0.014
IL-1β	0.646 (0.505–0.787)	0.052
IL-4	0.633 (0.491–0.776)	0.076
IL-10	0.612 (0.469–0.755)	0.137

AUROC, area under the receiver operating characteristic curve; CI, confidence interval; IL, interleukin; MCP, monocyte chemoattractant protein.

**Table 6:** Multivariate analysis of factors associated with increased hospital mortality

	Coefficient	Odds ratio (95% CI)	*P *value
APACHE II score (points)	0.313	1.37 (1.12–1.66)	0.002
Ln MCP-1	0.341	1.41 (1.02–1.93)	0.036
Constant	-8.335		

APACHE, Acute Physiology and Chronic Health Evaluation; CI, confidence interval; Ln, log transformed; MCP, monocyte chemoattractant protein.

## Discussion

Cytokine profiling of patients with severe sepsis may represent a valuable tool for delineating different patterns of immunological response, thus allowing identification of groups of patients with homogeneous biological derangements [6,24]. In the present study, a multiplex analysis of plasma cytokines in patients with severe sepsis and septic shock was able to identify cytokine profiles associated with early and late mortality, as well as evolution of organ dysfunction.

When we compared the cytokine profiles of septic shock patients with the profiles of patients with severe sepsis, we observed a significant increase in nine out of the 17 cytokines analyzed. Significant increases were observed in both proinflammatory and immunomodulatory cytokines. This included traditionally evaluated cytokines (IL-1β, IL-6, IL-8, IL-10 and TNF-α) and a number of cytokines that are not commonly associated with sepsis (IL-7, IL-13, IFN-γ and MCP-1). Moreover, for none of the cytokines evaluated was the concentration significantly lower in septic shock patients than in patients with severe sepsis.

It is increasingly recognized that the inflammatory response and deregulated cytokine production play key roles in the development of multiple organ dysfunction [5]. Clinically defined, early sequential analysis of organic dysfunction in severe sepsis has proven to be a good predictor of outcome [19,25]. However, the cytokine patterns associated with the evolution of organ dysfunction are not well established. A more restricted panel of cytokines (only six out of 17 cytokines), namely IL-1β, IL-6, IL-8, IL-10, MCP-1 and G-CSF, were found to correlate positively with organ dysfunction, as assessed by the SOFA score on day 1. Of these six cytokines, IL-8 and MCP-1 exhibited the best performance. In addition, concentrations of IL-6, IL-8 and G-CSF within the first 24 hours were predictive of worsening organ dysfunction or failure of organ dysfunction to improve on day 3. In contrast, although TNF-α concentrations failed to predict the evolution of organ dysfunction, or early or late mortality, TNF-α concentrations were significantly higher in patients with septic shock than in those with severe sepsis. These findings are in accordance with the recently proposed hypothesis that different patterns of cytokine profiles may be mirrored by distinct clinical presentations and severity [24].

Cytokines such as IL-6 and IL-8 are predictors of outcome in severe sepsis, a finding that is confirmed by our study; however, on multivariate analysis, they were not found to be independently associated with mortality. Interestingly, the best predictor of outcome in our study was MCP-1. The MCP-1 is a potent chemoattractant of mononuclear cells and a regulatory mediator in sepsis. Although its role is not entirely clear, its involvement in sepsis has been demonstrated during the past decade. Its pathophysiological role has been linked to the activated protein C pathway and its induced genes [26,27]. In animal models of sepsis, neutralization of MCP-1 was associated with significantly increased mortality [28,29]. Recently, our group demonstrated that endogenous MCP-1 positively regulates IL-10 but negatively controls macrophage migration inhibitory factor in experimental peritoneal sepsis, suggesting an important immunomodulatory role for MCP-1 in controlling the balance between proinflammatory and anti-inflammatory factors in sepsis [30]. In the clinical setting, only a few investigators have identified increased concentrations of MCP-1 in plasma [31] and bronchoalveolar lavage fluid [32] from septic patients, and those findings were not correlated with outcomes. Recently, Vermont and coworkers [33] reported that serum concentrations of MCP-1 in patients with meningococcal sepsis are predictive of mortality and correlate strongly with disease severity.

Because no single biomarker exhibits 100% accuracy in terms of predicting outcomes, it has been proposed that combinations of biomarkers and severity scores may yield better results. Oberholzer and coworkers [34] observed that IL-6 concentrations and APACHE II score were correlated, and that the combination of these variables exhibited good performance in predicting mortality in patients with severe sepsis. In the present study, among all evaluated cytokines, the combination of MCP-1 and APACHE II had the best accuracy.

Despite the fact that we were able to identify cytokines with good accuracy for predicting outcome in severe sepsis, our study has some limitations. The small sample size limits the extent to which our findings may be generalized to other groups of patients. Furthermore, only one time point was used for the measurement of cytokines, and although early evaluations are useful for entry criteria and early prognostic information, they do not allow one to derive further insights such as those provided by sequential measurement. However, cytokine concentrations on day 1 were associated with severity of organ failure on the first day and with failure of organ dysfunction to improve by day 3. Accordingly, both early and late mortality were also predicted. Although it is tempting to speculate that there is a direct correlation between cytokine concentrations and pathophysiology of organ injury, we believe one cannot attribute the full burden of disease severity to a particular cytokine. Cytokines may be increased simply as markers of tissue damage, without necessarily playing a direct role.

The multiplex system provides the opportunity to establish a panel of sepsis biomarkers that could include not only currently evaluated cytokines that have diagnostic/prognostic value but also other valuable sepsis biomarkers, such as including migration inhibitory factor [35], procalcitonin [36] and triggering receptor expressed on myeloid cells-1 [37]. We fully acknowledge that the complexity of sepsis requires new perspectives if we are to achieve an integrated understanding of the intricate interactions that occur during the disease process, rather than describing isolated aspects of it [38].

## Conclusion

Simultaneous evaluation of multiple cytokines in early severe sepsis may reveal cytokine patterns that reflect the inflammatory response associated with evolution of organ dysfunction as well as early and late mortality. A knowledge of the cytokine profiles associated with distinct clinical presentations and outcomes may be useful in the design of future studies of biomarkers in sepsis that involve larger patient populations.

## Key messages

• Simultaneous analysis of multiple cytokines proved useful in identifying cytokine patterns of inflammatory response associated with evolution of organ dysfunction as well as early and late mortality in patients with severe sepsis and septic shock.

• Among the 17 cytokines evaluated, IL-8 and MCP-1 exhibited the best correlation with organ dysfunctions on day 1; in addition, IL-6, IL-8 and G-CSF concentrations within the first 24 hours were able to predict worsening organ dysfunction or failure of organ dysfunction to improve by day 3.

• In terms of predicting mortality, the cytokines IL-1β, IL-4, IL-6, IL-8, MCP-1 and G-CSF had good accuracy for predicting early mortality (< 48 hours), and IL-8 and MCP-1 had the best accuracy for predicting 28-day mortality; in the multivariate analysis only MCP-1 was independently associated with prognosis.

## Abbreviations

APACHE = Acute Physiology and Chronic Health Evaluation; AUROC = area under the receiver operating characteristic curve; CI = confidence interval; ELISA = enzyme-linked immunosorbent assay; G-CSF = granulocyte colony-stimulating factor; IFN = interferon; IL = interleukin; MCP = monocyte chemoattractant protein; ROC = receiver operating characteristic; SOFA = Sequential Organ Failure Assessment; TNF = tumour necrosis factor.

## Competing interests

The authors declare that they have no competing interests.

## Authors' contributions

FAB contributed to the study conception and design, carried out clinical studies, and participated in data analysis and drafted the manuscript. JIS and AMJ carried out the clinical studies and participated in the data analysis. EFA and RNG carried out the Luminex immunoassays and participated in the data analysis. MS performed the statistical analysis. MTB, HCFN and PTB conceived the study, and participated in its design and coordination, supervised data analysis and helped to draft the manuscript. All authors read and approved the final manuscript.
